# 3D microgroove electrical impedance sensing to examine 3D cell cultures for antineoplastic drug assessment

**DOI:** 10.1038/s41378-020-0130-x

**Published:** 2020-03-09

**Authors:** Yuxiang Pan, Deming Jiang, Chenlei Gu, Yong Qiu, Hao Wan, Ping Wang

**Affiliations:** 1 Biosensor National Special Laboratory, Key Laboratory for Biomedical Engineering of Education Ministry, Department of Biomedical Engineering, Zhejiang University, Hangzhou, 310027 China; 2 State Key Laboratory of Transducer Technology, Chinese Academy of Sciences, Shanghai, 200050 China

**Keywords:** Biosensors, Nanostructures

## Abstract

In recent decades, three-dimensional (3D) cancer cell models have attracted increasing interest in the field of drug screening due to their significant advantages in more accurate simulations of heterogeneous tumor behavior in vivo compared to two-dimensional models. Furthermore, drug sensitivity testing based on 3D cancer cell models can provide more reliable in vivo efficacy prediction. The gold standard fluorescence staining is hard to achieve real-time and label-free viability monitoring in 3D cancer cell models. In this study, a microgroove impedance sensor (MGIS) was specially developed for the dynamic and noninvasive monitoring of 3D cell viability. 3D cancer cells were trapped in microgrooves with gold electrodes on opposite walls for in situ impedance measurement. The change in the number of live cells caused inversely proportional changes to the impedance magnitude of the entire cell/Matrigel construct and reflected the proliferation and apoptosis of the 3D cells. It was confirmed that the 3D cell viability detected by the MGIS was highly consistent with the standard live/dead staining by confocal microscope characterization. Furthermore, the accuracy of the MGIS was validated quantitatively using a 3D lung cancer model and sophisticated drug sensitivity testing. In addition, the parameters of the MGIS in the measurement experiments were optimized in detail using simulations and experimental validation. The results demonstrated that the MGIS coupled with 3D cell culture would be a promising platform to improve the efficiency and accuracy of cell-based anticancer drug screening in vitro.

## Medical sensors: 3D cancer cell analysis

A new micro groove impedance sensor enables the real-time and high-throughput analysis of 3D lung cancer cell models. In-vitro cell-based assays are important for the screening of new cancer drugs. However, many drug screening approaches only work for 2D arrangements of cells, yet it is known that 3D arrangements can behave very differently. Electrochemical impedance spectroscopy is widely used for monitoring the behavior of 2D arrangements of cells, yet attempts to extend it to 3D are limited. Now, a team led by Ping Wang from Zhejiang University demonstrate a micro groove impedance sensor for drug screening of 3D cancer cell models. Substantial differences were seen compared to 2D analysis, demonstrating the importance of tools for assessing 3D cancer models in drug development.

## Introduction

Among the various challenges in medicine, the cure for cancer has always been the hardest and most remarkable. The era of chemotherapy began in the 1940s with the first use of nitrogen mustards and antifolate drugs^1^. Despite the industrialization of drug screening and the great deal of money and resources invested in new drug development each year, most of these drug developments fail due to inefficiency and unpredictable side effects using conventional drug screening methods such as animal testing^2^. In vitro cell-based assays are an alternative drug screening method, featuring fast, low cost, and high throughput compared to conventional animal testing^3^. However, the two-dimensional (2D) cells used in traditional cell-based assays are generally cultured on flat and rigid substrates, thus exhibiting different features from in vivo cells in terms of morphological structure, heterogeneity, and intercellular connectivity. Occasionally, 2D cell-based drug screening may provide conflicting or misleading conclusions^4^. Recently, three-dimensional (3D) cancer cell models have attracted increasing interest due to their significant advantages in simulating in vivo heterogeneous tumor behavior in a more accurate manner compared to 2D models^5^. Furthermore, it has been demonstrated that 3D cells are able to provide more precise cellular responses to drug candidates in a setting that resembles in vivo environments^6,7^.

Chemotherapeutic drugs are capable of curing cancer by accelerating tumor cell death^8^. Therefore, by monitoring the viability of 3D cancer cells, the drug efficacy for cancer treatment can be effectively evaluated. However, conventional methods for detecting cell viability, such as the MTT (3-[4,5-dimethylthiazole-2-yl]-2,5-diphenyltetrazolium bromide) assay and CCK-8 (Cell Counting Kit-8) assay, can only be applied to 2D cells cultured in a highly fluid medium solution^9^. Live/dead (L/D) cell staining (calcein-AM/propidium iodide (PI) assay) is an endpoint detection method that provides very limited data and fails to monitor the temporal and spatial activity of 3D cells^10,11^. High-resolution confocal fluorescence microscopy is generally utilized for the quantitative analysis of 3D cells with accurate morphological characterization considering these spatial and physical traits in 3D cultures^6,12^. However, it also suffers from being time-consuming and labor-intensive and fails to meet the high-throughput requirement of drug screening^3^.

To counteract these methodological limitations, electrochemical impedance spectroscopy (EIS) has been widely applied for noninvasive, real-time, high-throughput analysis of cell viability, proliferation, and cytotoxicity^13,14^. Based on the EIS technique, electric cell-substrate impedance sensing (ECIS) has been used for real-time and label-free monitoring of 2D cell behaviors, such as cell viability, proliferation, and apoptosis^15–17^. The impedance changes detected by ECIS are determined by the viability and number of cells attached to 2D planar interdigitated electrodes (IDEs)^18^. However, 3D cells are encapsulated in poorly conductive Matrigel and cannot directly attach to planar IDEs, which hinders the monitoring of 3D cell viability using traditional ECIS technologies^19^.

In this study, we specifically designed a multidimensional microgroove impedance sensor (MGIS) for real-time, noninvasive, and high-throughput pharmacokinetic analysis of chemotherapeutic candidates using 3D lung cancer models. To overcome the technical limitations of traditional 2D ECIS, a microgroove structure was designed with multiple electrodes to trap 3D cancer cells for 3D ECIS. The goal of 3D ECIS is to improve the predictability and reduce the false-positive rate of drug testing in vitro. Moreover, an innovative 3D cell electrical model and equivalent circuit were established for in-depth cellular behavioral analysis. The stability, electrical properties, reproducibility, and long-term reliability of the MGIS were tested to validate the sensor performance. A 3D lung cancer model was constructed using 3D cell culture, and this work, for the first time, compared a 3D lung cancer model with traditional 2D cells to evaluate the transferability of cell model data to the in vivo situation for lung cancer drug screening. The MGIS combined with 3D ECIS can provide efficient and accurate in vitro data similar to in vivo cells, which can establish a promising platform for accurate drug efficacy evaluation and effective personalized treatment.

## Materials and methods

### Detection principle of 3D ECIS using the MGIS

**Fig. 1:**
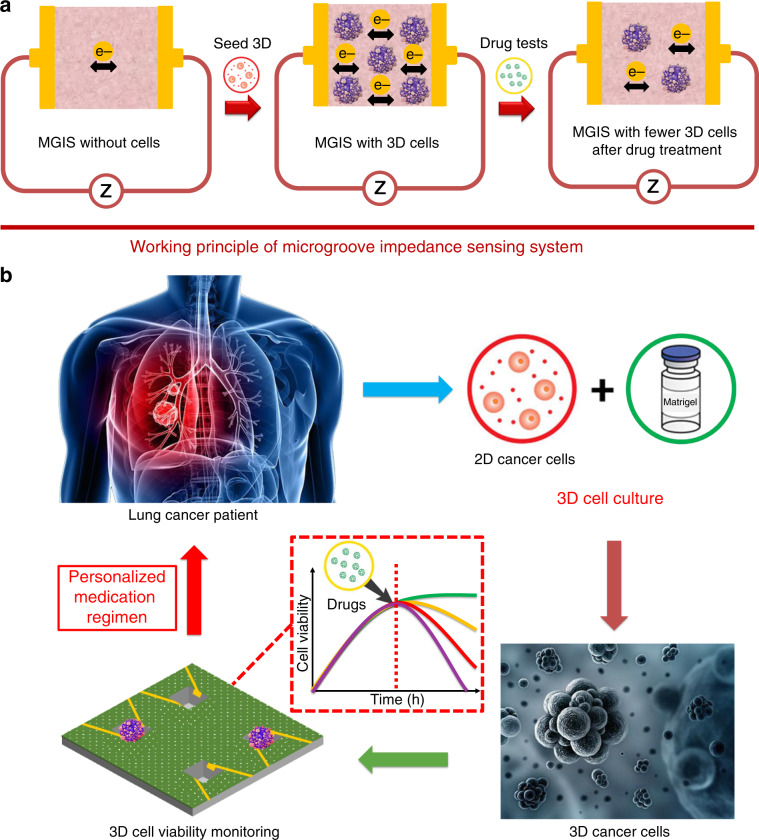
Schematic illustration of 3D cell sensing and detection. **a** The working principle of 3D ECIS: the number of living cells influences the conductivity of the Matrigel/cell construct. **b** Schematic of the 3D ECIS for antineoplastic drug screening

The traditional EIS technique for 2D cell viability monitoring requires the attachment of cells onto the sensor surface. However, 3D cells are encapsulated and cultured in a Matrigel that prevents the movement and attachment of 3D cells. Therefore, the 3D ECIS principle for 3D cell monitoring is discussed in this study. It has been observed that the conductivity of the Matrigel increases after live cells are mixed into the Matrigel, although Matrigel is often considered a nonconductor with a high initial impedance^20^. This result indicates that live cells can promote ions passing to the electrodes. In general, a single cell is considered a nonconductor due to the lack of conductivity of the cell membrane^21^. However, intercellular communication is achieved by gap junctions that serve as channels on the cell membrane and enable electrical connections to neighboring cells^22^. When an external electrical field is applied to the cells, the gap junctions between cells form electrical connections^23^, thereby causing an increase in the conductivity of the cell/Matrigel construct that is directly related to the number of cells in the construct. When the cells in the construct undergo apoptosis after drug treatment, the number of gap junctions decreases, causing an increase in the impedance of cell/Matrigel (Fig. 1a). In the above-referenced study, the cell index (CI) was introduced to normalize the impedance values for data analysis. Here, CI is defined as the ratio of the 3D cell growth-induced impedance change I∆*Z*I to the background impedance (*Z*0)^17^, that is, CI = I∆*Z*I/*Z*0. Based on the above principles, 3D cell activity, proliferation, and apoptosis analysis can be achieved by 3D ECIS monitoring of the overall impedance change ratio of the 3D cell/Matrigel construct (Fig. 1b).

### Fabrication of the MGIS

**Fig. 2:**
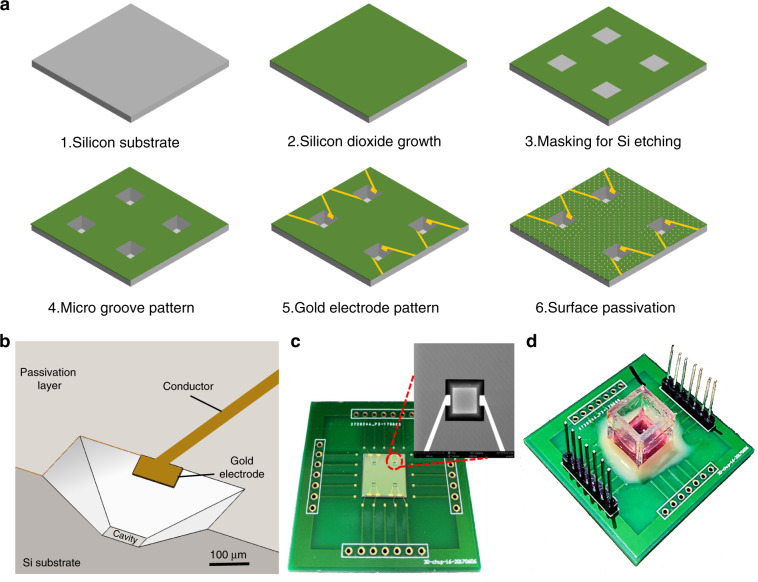
Construction of 3D MGIS chip. **a** Fabrication scheme of the silicon-based multidimensional microgroove impedance sensor. **b** Cross-sectional scheme of the microgroove structure for trapping 3D cells and in situ impedance measurement. **c** Photo of the MGIS chip. Inset: SEM image of a square microgroove (800 μm × 800 μm) in the silicon substrate with a pair of opposite gold electrodes. **d** Photograph of the complete MGIS chip with 3D cell culturing

The MIGS chip was fabricated using standard microelectromechanical system (MEMS) technologies (Fig. 2a). A 4-in. silicon wafer was used for the optimum yield of the MGIS chips, where each chip has dimensions of 10 mm × 10 mm × 0.5 mm. First, the silicon wafer was thermo-oxidized in an O_2_ atmosphere at 1050 °C to obtain a 1500 nm SiO_2_ layer over the entire wafer surface. Second, the silicon dioxide layer was etched into several edge lengths of 800 μm square shapes by a chrome mask and lithography using hydrofluoric acid solution. Then, the microgroove structure was realized by wet chemical anisotropic etching in a 40% potassium hydroxide solution at 90 °C. The square holes were etched into the silicon substrate at an angle of 54.7° according to the crystal orientation of silicon (1 0 0). The depth of the microgroove structure was determined by the etching time; for application purposes, 100 μm was chosen as the optimal depth. Afterwards, the structured wafer was subjected to secondary thermal oxidation to obtain a 1500 nm SiO_2_ coating to improve subsequent electrical insulation. Third, the structured wafer surface was spin coated with photoresist S1813 at 4000 r.p.m. for 45 s and soft-baked on a hot plate at 115 °C for 1 min. The wafer was then exposed to ultraviolet (UV) for electrode patterning and developed by developer 352. After careful cleaning by using deionized water and N_2_, 10 nm titanium combined with 500 nm gold was sputtered on the patterned wafer as the metal layer. The electrodes on the sidewalls of the cavities and chip conductor paths were realized by a lift-off process. To overcome the problem of fabricating electrodes on the sloped sides, a full lift-off process was conducted by 2 h of acetone immersion and ultrasonic cleaning. Subsequently, a silicon nitride layer (Si_3_N_4_, 700 nm) was deposited onto the wafer surface as the passivation layer by plasma-enhanced chemical vapor deposition at 350 °C with SiH_4_ and NH_3_. To implement 3D ECIS for 3D cells, the working electrodes on the sidewalls of the microgroove were bared by a reactive ion-etching process on an STS 320 with CHF_3_ and CF_4_ (Fig. 2b). Finally, the batch-processed wafers were sawed into 60 individual MGIS chips, where each chip had four microgrooves with a uniform depth of 100 μm and a top edge length of 800 μm (Fig. 2c). After the chip was electrically connected to an adapter board by wire bonding, a polymethyl methacrylate culture chamber with good biocompatibility was fixed onto the MGIS chip by using Henkel Loctite HY4090 structural hybrid adhesive (Fig. 2d).

### 3D cell culture

Adenocarcinomic human alveolar basal epithelial cells (A549, American Type Culture Collection) were maintained in RPMI (Roswell Park Memorial Institute)-1640 medium (Gibco, USA) with 10% fetal bovine serum (FBS, Gibco, USA) and 1% penicillin–streptomycin (Gibco, USA). Human hepatoma cells (HepG2) and Madin–Darby Canine Kidney cells (MDCK) (American Type Culture Collection) were cultured in Dulbecco’s modified Eagle’s medium (DMEM, Gibco, USA) with 10% FBS (Gibco, USA) and 1% penicillin–streptomycin (Gibco, USA). A549, HepG2, and MDCK cells were placed in a 37 °C and 5.0% CO_2_ humidified incubator (Thermo Fisher Scientific, USA) for 2D culture, and the medium was renewed every 2 days. When the 2D cells reached 90% confluence, 0.25% trypsin/EDTA solution (Gibco, USA) was used to passage cells for 3D culture. The construction of the 3D cancer models was implemented according to Lee’s protocol^24^. The detached cells were transferred to a centrifuge tube and mixed with prechilled Matrigel solution (1:1, BD Biosciences, USA) to obtain a cell suspension with a cell density of 5 × 10^6^/mL. Two hundred microliters of the Matrigel/cell mixture was added to the MGIS chip and gelated at 37 °C in a 5.0% CO_2_ humidified incubator. After 30 min of heat curing, 100 μL of medium was added to the MGIS chip for nutritional support.

### Chemotherapeutic treatments

The broad-spectrum antitumor drug cisplatin (Solarbio, China) was used to assess the capability of the MGIS with a 3D lung cancer model for drug efficacy evaluation. When the 2D and 3D cells reached the growth plateau, 10, 100, and 1000 μM cisplatin were added to the medium for the cytotoxicity study. Analysis of drug synergy was performed by the pharmacological intervention of 10 μM cisplatin, 10 μM cisplatin + 10 μM gemcitabine (Solarbio, China), and 10 μM cisplatin + 10 μM pemetrexed (Solarbio, China). All drug testing protocols were based on the NCCN guidelines for lung cancer treatment^25^.

### 3D ECIS measurements

First, AC impedance measurement was conducted by a CHI 660e electrochemical analyzer (CH Instruments, China) to determine the optimal detection frequency for the MGIS. An initial electrical potential of 50 mV was applied to the working electrodes on the walls of the microgroove, and the impedance was measured in the range of 1000 Hz to 100 kHz to determine the optimal frequency for maximum relative impedance. By applying the optimal frequency, the viability of 3D cells can be measured by the impedance value change ratio. To achieve full evaluation of 3D cell cultures and drug efficacy, real-time impedance monitoring of the 3D cells was conducted for 7 days.

### Calcein-AM/PI staining

To verify the consistency of the 3D cell viability monitored by the MGIS chip with the gold standard method, we adopted a Calcein-AM/PI staining Double Stain Kit (Dojindo, Japan) to characterize the L/D assessment of the 3D cells. L/D staining was performed on days 0, 3, and 7. One microliter of 1 mol/L calcein-AM (live cell staining dye) and 1.5 mol/L PI (dead cell staining dye) were mixed into a cell culture dish containing 1 mL of medium. Then, the mixture was incubated in the dark at 37 °C for 30 min. The medium was flushed from the cell culture dish manually using phosphate-buffered saline three times. The 3D cells were imaged in situ by using a confocal microscope (Olympus FV1000, Japan) at ×100 magnification. Z-stacks of 100 μm were taken of each 3D cell construct, from which maximum projections (2D compressed images) and 3D reconstructed images were obtained. The 3D cell viability was defined by ImageJ (National Institutes of Health, USA) as the percentage of viable cells (green) in the maximum 2D compressed image relative to the total number of cells (green and red).

### Statistical analysis

All statistical analysis was performed with GraphPad Prism 6.0 (GraphPad software, USA). All the data are presented as the means ± standard deviations for at least three repeats. Comparisons between two groups were analyzed by Student’s *t* test (two tailed) with two-sample unequal variances, and *p* < 0.05, 0.01, and 0.001 were considered significant (*), very significant (**), and highly significant (***), respectively.

## Results and discussion

### Electrical simulation and optimization of the 3D ECIS

**Fig. 3:**
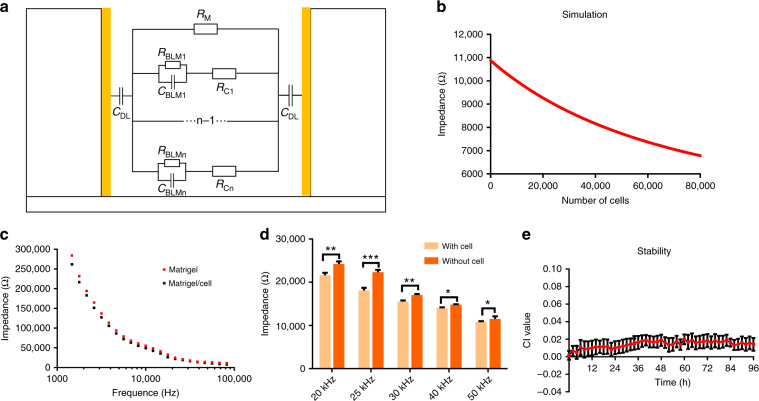
Electrical simulation and optimization of the 3D MGIS. **a** Equivalent circuit of the Matrigel/cell construct in the microgroove. **b** Simulation impedance with different numbers of cells. **c** Bode plot of the Matrigel/cell construct and the construct without cells. **d** Optimization of the detection frequency. **e** Stability analysis of the MGIS chip, which was conducted by long-term testing of the Matrigel. All the data are represented by the means ± SDs (standard deviations), *n* = 3

Traditional ECIS techniques require good attachment of 2D cells to the IDE surface. However, since 3D cells are encapsulated in Matrigel, the equivalent circuit for 3D ECIS is quite different from that of traditional ECIS for 2D cells. For impedance analysis in 3D cells, an equivalent circuit model consisting of a double layer capacitance between the electrolyte and the electrode (*C*_DL_), the resistance of the Matrigel (*R*_m_), the capacitance of the cell membrane (*C*_BLM_), and the resistance of the cell (*R*_c_) was designed based on Morgan’s theory (Fig. 3a)^26^. Our previous studies verified the rationality of this model. It was found that the impedance of the construct decreased with increasing frequency^19^, which was consistent with the actual test result. In the measurement of 3D cell viability, the total impedance of the cell/Matrigel construct can be divided into the Matrigel impedance portion and cell impedance portion. Since the impedance of the Matrigel remains relatively constant during the measurement, the total impedance change is mainly determined by the cell impedance portion, which depends on the number of living cells. A larger number of living cells would lead to more gap junctions and greater conductivity in the Matrigel/cell construct. On the basis of our previous studies, we simulated the correlation between cell viability and impedance at 25 kHz using MATLAB (R2012a, MathWorks, USA). The exact value of each electrical component was calculated with the following equations:

$$\begin{array}{l}Z_{\mathrm{wo}} = \frac{1}{{j2\pi fC_{\mathrm{DL}}}} + R_{\mathrm{m}},\\ Z_{\mathrm{w}} = \frac{1}{{j2\pi fC_{\mathrm{DL}}}} + \frac{1}{{\frac{1}{{R_{\mathrm{m}}}} \,+\, \frac{1}{{R_{\mathrm{ce}} \,+\, \frac{1}{{j2\pi fC_{\mathrm{ce}}}}}}}},\end{array}$$

where *Z*_w_ and *Z*_wo_ are the measured impedances with and without cells, respectively, *j* is the imaginary unit, *f* is the detecting frequency, and *R*_ce_ and *C*_ce_ are the equivalent resistance and capacitance, respectively, of *R*_BLM_, *C*_BLM_, and *R*_c_. It was found that an increase in the number of living cells led to a decrease in the impedance, which is highly consistent with the results of the MGIS chip in the real-time monitoring of cell viability (Fig. 3b). Therefore, based on this electrical model, the 3D cell pharmacodynamic response can be predicted from the data measured by the MGIS chip.

To obtain the largest impedance change ratio, the detection frequency of the MGIS chip was optimized. EIS was performed in the range of 1000 Hz to 100 kHz. Figure 3c displays the impedance values versus different frequencies measured by the 3D ECIS. The impedance of the 3D cells tended to decrease with increasing detection frequency. The impedance of the Matrigel/cell construct at 25 kHz remarkably decreases, as shown in Fig. 3d (*n* = 3, ***p < 0.001). This result indicates that 25 kHz is the optimal frequency for achieving significant experimental results in chemotherapeutic treatment tests. On the other hand, the stability of the MGIS chip has a great impact on the accuracy of the detected data, and so a long-term stability test was conducted on the MGIS chip. As shown in Fig. 3e, the impedance value of the Matrigel without cells after inoculation into the MGIS chip remained stable for a long time.

### Long-term monitoring of 3D cell viability

**Fig. 4:**
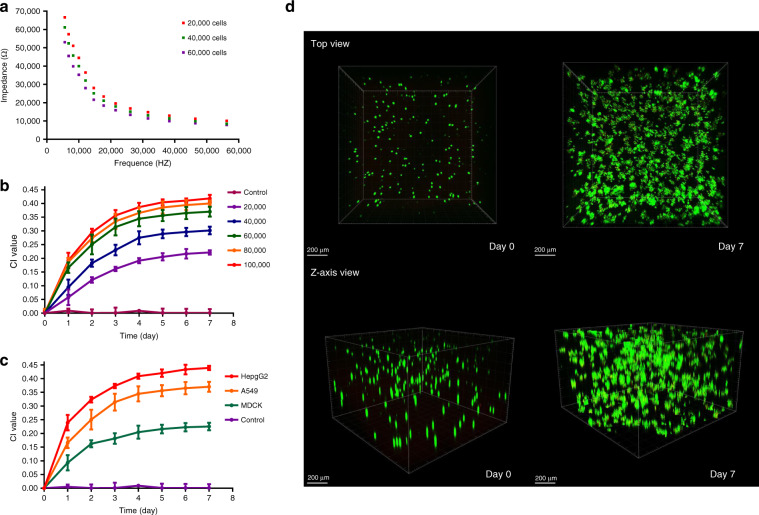
Confirmation of 3D MGIS for long-time monitoring of 3D cell viability. **a** Bode plot of the Matrigel/cell construct with cells at different seeding densities. **b** A group of representative cell growth curves for 3D A549 cells with different numbers of cells ranging from 20,000 to 100,000 cells/well. **c** Representative cell growth curves of 3D cultured A549 cells, HepG2 cells, and MDCK cells. **d** Live/dead analysis on days 0 and 7 of the Matrigel/cell constructs. The top image represents the top view of the total construct; the bottom image represents a 3D reconstruction of a 100 μm Z-stack of confocal images. Green fluorescence indicates calcein-AM-stained live cells, and red fluorescence indicates PI-stained dead cells; scale bar: 200 μm. All the data are represented by the means ± SDs (standard deviations), *n* = 3

L/D staining is the gold standard method for 3D cell viability monitoring, but it has difficulty in meeting the needs of noninvasive and high-throughput detection. The above-mentioned MGIS chip can accurately distinguish the impedance changes in the construct with or without cells. Here, we conducted an in-depth analysis of the impedance changes with different numbers of cells. The A549 cell line was used to study the differences in the construct impedance at different cell densities. As shown in Fig. 4a, the MGIS chip can effectively distinguish the construct impedance with different cell densities, which is consistent with the theoretical modeling. Lung cancer cell growth curves with different cell seeding densities were also obtained after culturing for 7 days. The growth curve for 60,000 cells/well is stable and reaches the growth plateau faster, which is beneficial for the subsequent drug testing experiments (Fig. 4b). Therefore, we chose 60,000 cells/well as the inoculation density of the 3D lung cancer model. To verify the applicability and reproducibility of the MGIS chip, we adopted another two cell lines (HepG2 and MDCK) to verify the sensor performance. It was found that different types of cells produced different growth curves with the same initial seeding density (Fig. 4c). The results are consistent with the kinetics of cell growth in that different types of cells have different proliferation abilities and reach a plateau at different times. To verify the accuracy of the growth data measured by the MGIS chip, we used fluorescence confocal imaging to characterize the 3D A549 cells at 0 and 7 days. The images in Fig. 4d show that the cell density of the 3D cells at day 7 obviously increased compared to that at day 0. Therefore, it can be concluded that 3D ECIS can be used for the long-term monitoring of 3D cell activity and proliferation.

### Real-time monitoring of cisplatin gradient effects using the 3D lung cancer model

**Fig. 5:**
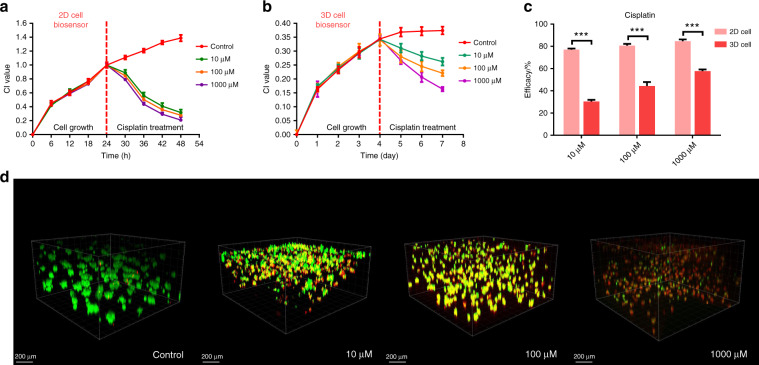
Real-time monitoring of cisplatin gradient effects on 3D lung cancer model. **a** The normalized cell growth curves of 2D A549 cells responding to different concentrations of cisplatin in ECIS. **b** The normalized cell growth curves of 3D A549 cells responding to different concentrations of cisplatin in 3D ECIS. **c** Differential analysis of 2D ECIS and 3D ECIS for measuring cisplatin efficacy. **d** L/D analysis on day 7 of constructs exposed to the indicated cisplatin concentration. Each image represents a 3D reconstruction of a 100 μm Z-stack of confocal images taken at the conclusion of the experiment (day 7). All the data are represented by means ± SDs (standard deviations), *n* = 3

Apoptosis of 3D cells decreases the number of gap junctions and further leads to an increase in the impedance of the 3D cell/Matrigel construct. Based on this principle, we further explored the ability of 3D ECIS to monitor 3D cell apoptosis. A stable 3D lung cancer model was established using the previously optimized cell density for apoptosis experiments in antineoplastic drug testing. The common lung cancer chemotherapy drug cisplatin can bind to DNA and cause cross-linking, thereby destroying DNA function and inhibiting cell mitosis^27^. Three concentrations of cisplatin (10, 100, and 1000 μM) were added to 2D A549 cells and 3D A549 cells that had reached the growth plateau. As shown in Fig. 5a, all three concentrations of cisplatin had a good effect on the 2D cells. However, ECIS based on 2D cells has difficulty distinguishing the pharmacodynamic differences of three concentrations of cisplatin. Conversely, 3D ECIS can distinguish the effects of the three concentrations of cisplatin on the viability of the 3D lung cancer models. The highest concentration of cisplatin had the largest effect on the CI value of the 3D cells (Fig. 5b). The maximum drug efficacy of all tested concentrations of cisplatin was compared among the lung cancer models, and highly significant differences were observed between 2D ECIS (10 μM: 77.11 ± 0.58%; 100 μM: 80.67 ± 0.88%; 1000 μM: 84.69 ± 0.89%) and 3D ECIS (10 μM: 30.44 ± 0.87%; 100 μM: 44.38 ± 2.03%; 1000 μM: 57.73 ± 0.89%) (Fig. 5c). The reason for this is that 2D cells lack an extracellular matrix and the intercellular connections that occur in the 3D in vivo environment, and anticancer drugs can work directly on fragile monolayer cells. As 3D cells have a natural shape in spheroid/aggregate structures, drugs may not be able to fully penetrate the spheroid and cannot reach the cells near the core. Consequently, 2D cells often succumb to treatment with even low concentrations of drugs. Therefore, the ability of 2D cells to distinguish the efficacy of different concentrations of antitumor drugs is inferior to that of 3D cells. These results also confirmed that the in vitro 2D cell assay may sometimes produce false-positive results, which cannot accurately reflect the results of the 3D cell assay and in vivo experiments. Additionally, we used fluorescence confocal experiments to verify the accuracy of the cell apoptosis data measured by the MGIS chip. As shown in Fig. 5d, different concentrations of cisplatin have different cell apoptosis effects on 3D A549 cells, which is consistent with the results measured by 3D ECIS. A number of studies have found that 3D cellular responses to drug treatments are more similar to in vivo responses than those of 2D cells and that 3D cells are more resistant to anticancer drugs than 2D cells^28^. For example, ovarian cancer cells survived 40% or 60% less frequently in 3D cells after paclitaxel treatment; however, the same treatment resulted in an 80% reduction in cell survival in 2D cells monolayers^29^. Engineering personalized tumor ecosystems based on 3D cell culture were applied to predict the clinical response in an independent validation group of 55 patients and showed high sensitivity in predictions^30^. These results validated that 3D ECIS could be an effective tool for the prediction of anticancer effects in vivo.

### Correlation of 3D ECIS and standard assay data of single and combined antineoplastic drugs

Since cancer cells can easily adapt to targeted therapies, current cancer treatment strategies focus primarily on the combined use of multiple drugs to avoid drug resistance^31^. In this study, 3D ECIS was used to analyze the efficacy of combination therapy versus a single drug in a 3D lung cancer model. Since cell viability assay methods such as fluorescent staining have been widely used in in vitro drug development, 3D ECIS analysis should also yield similar results in the field of drug development.

**Fig. 6:**
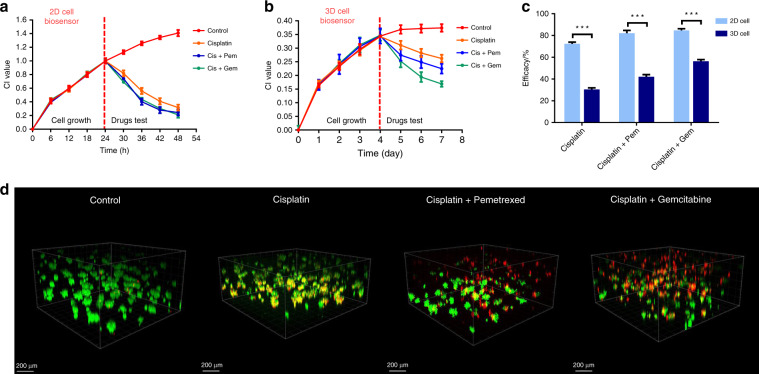
Evaluation of the susceptibility of 3D cells with different drug combinations. **a** The normalized cell growth curves of 2D A549 cells responding to different combinations of antineoplastic drugs on 2D ECIS. **b** The normalized cell growth curves of 3D A549 cells responding to different combinations of antineoplastic drugs on 3D ECIS. **c** Differential analysis of the efficacy of the three drug combinations for 2D ECIS and 3D ECIS. **d** L/D analysis on day 7 of constructs exposed to the indicated drug combinations. Each image represents a 3D reconstruction of a 100 μm Z-stack of confocal images taken at the conclusion of the experiment (day 7). All the data are represented by the means ± SDs (standard deviations), *n* = 3

To verify the correlation of 3D ECIS and the standard assay, three chemotherapeutic regimens were used for cytotoxicity experiments. Analysis of drug synergy was achieved by pharmacological intervention with 10 μM cisplatin, 10 μM cisplatin + 10 μM gemcitabine, and 10 μM cisplatin + 10 μM pemetrexed. Gemcitabine and pemetrexed are two common lung cancer combination ligands that can disrupt the cell cycle and promote apoptosis, which can enhance the efficacy of cisplatin according to clinical trial reports^32,33^. As shown in Fig. 6a, ECIS based on 2D cells cannot distinguish the efficacy of monotherapy and combination therapy, and the cell growth curves of the three groups of drugs show slight differences. In contrast, the curves obtained by the 3D ECIS effectively show that the efficacy of cisplatin is enhanced by the two synergistic drugs (Fig. 6b). Significant differences were observed between the maximum efficacy of the three chemotherapy regimens measured in 2D ECIS and 3D ECIS. Highly significant differences can be observed between 2D ECIS (cisplatin: 72.32 ± 0.88%; cisplatin + pemetrexed: 82.07 ± 1.53%; cisplatin + gemcitabine: 84.62 ± 0.87%) and 3D ECIS (cisplatin: 30.34 ± 0.89%; cisplatin + pemetrexed: 42.17 ± 1.16%; cisplatin + gemcitabine: 56.39 ± 0.92%) (Fig. 6c). These results indicate that the three combinations are all highly active in 2D monolayer cells, but generally less active and have different effects in 3D cells. The reason for this is that the combination of drugs can enhance the killing ability of a single drug against tumors, but the synergistic effect of different drugs is different. 3D cells are often more resistant to treatment than 2D cells and can thus be more accurate predictors of in vivo drug responses. As shown in Fig. 6d, the results of fluorescence confocal characterization are highly consistent with the data obtained by the 3D ECIS. In fact, traditional quantitative analysis of 3D cells relies on confocal fluorescence microscopy, which has great spatial resolution and optical sectioning capabilities. However, due to irreversible damage to the cells caused by fluorescent dyes, fluorescent staining can only provide limited and static data for the kinetics of cellular responses. 3D ECIS can provide dynamic and long-term viability data for 3D cells in a noninvasive and label-free way. The above results demonstrate that 3D ECIS can adapt to drug screening based on the MGIS, which can effectively resolve the issue of the high false-positive rate in conventional 2D cell-based biosensors.

## Conclusions

In this study, we developed a real-time, noninvasive, and high-throughput MGIS for antineoplastic drug assessment based on 3D cancer cell models. The close correlation of L/D staining with the pharmacokinetic results obtained by EIS proved that the real-time, noninvasive 3D ECIS was of great value for the pharmacological screening of short/long-term single-agent and combination drugs. In addition, the substantial differences in the drug responses between the in vivo and in vitro 2D cell assays highlight the importance of 3D cancer models in drug development. The clinically observed effects of the single-agent and combination therapies on nonsmall-cell lung cancer are very similar to the results of this study.

To better achieve precision medicine, organoid technology should be used to more accurately simulate in vivo solid tumors and coupled with the MGIS chip to achieve organoid-based biosensors for pharmaceutical screening. The prototype of the microgroove impedance sensor has been extended to a 48-channel impedance analysis platform for standardized and parallel analyses of cancer therapeutics on 3D cancer models or organoids.
